# Supplementary material for: Induced organoids derived from patients with ulcerative colitis recapitulate colitic reactivity
Source: Nat Commun. 2021 Jan 11;12:262. doi: 10.1038/s41467-020-20351-5 (PMC7801686; doi:10.1038/s41467-020-20351-5)
Supplement: Supplementary file 1 — Supplementary Information [file 41467_2020_20351_MOESM1_ESM.pdf]

Supplementary Figure 1 related to Figure 1

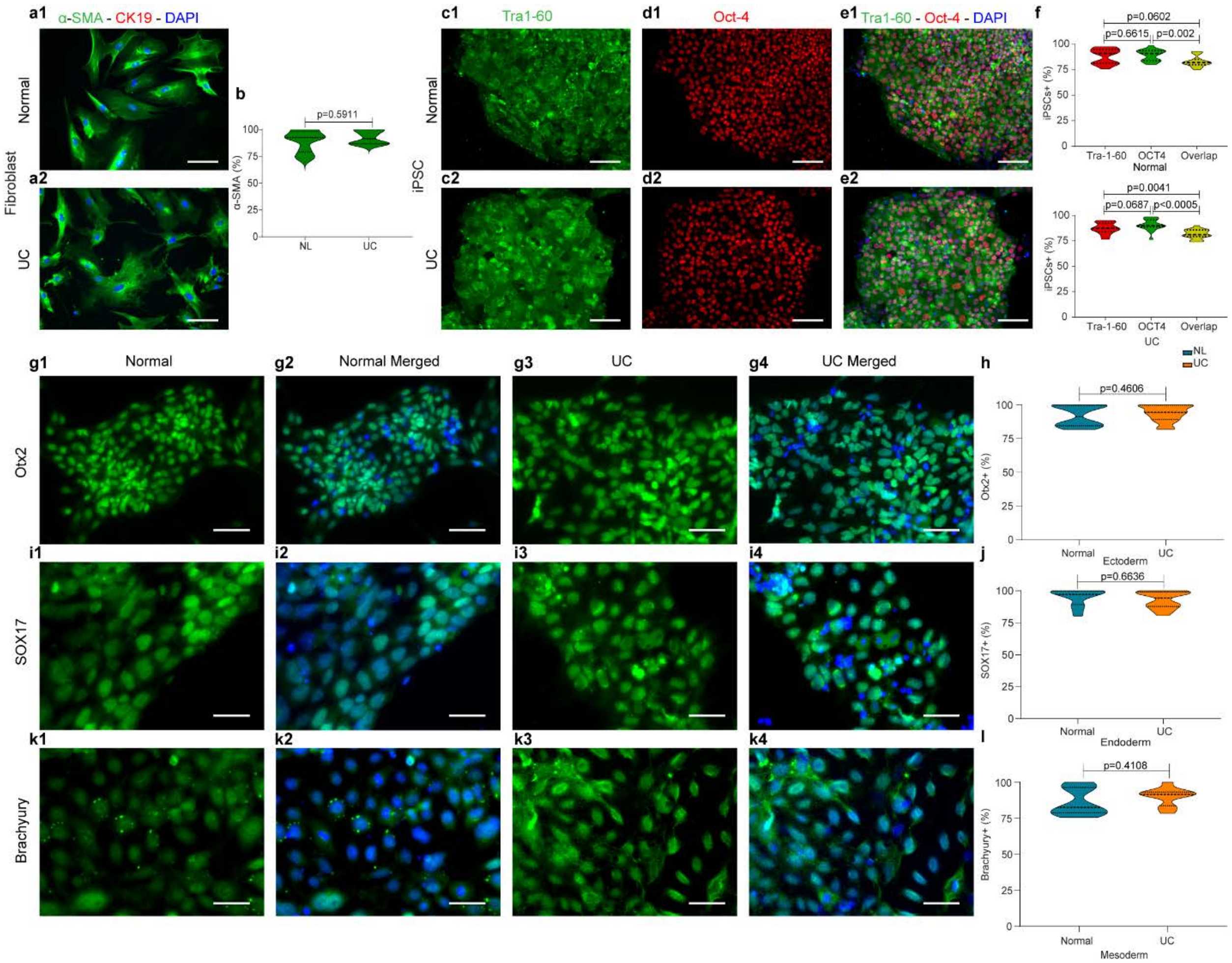

Supplementary Fig. 1: Confirmation of pluripotency in induced pluripotent stem cells

a Representative IF staining to demonstrate α-SMA (green) expression and absence of CK19 (red) in normal and UC parental fibroblasts. **b** Violin plots showing the distribution of the cells expressing α-SMA and CK19 in normal and UC fibroblasts. **c, d, e** Representative dual IF staining for Tra-1-60 (green) and Oct-4 (red) pluripotent markers in normal and UC iPSCs. **f** Violin plots for the iPSCs expressing Tra-1-60, Oct-4, or both. **g, i, k** Representative IF staining for Otx2 (green), SOX17 (green), and brachyury (green), confirming the differentiation of the iPSCs to ectoderm, endoderm, and mesoderm, respectively. **h, j, l** Percentage distribution of the cells expressing Otx2, SOX17, and brachyury, respectively. Nuclei in all IF images were counterstained for DAPI (blue). An unpaired, non-parametric, two-sided Mann-Whitney *U* test was used for all statistical comparisons. Scale bar= 25 μm. n=5 iHNOs and n=6 iHUCOs. Source data are provided as a Source Data file.

Supplementary Figure 2 related to Figure 1 & 2

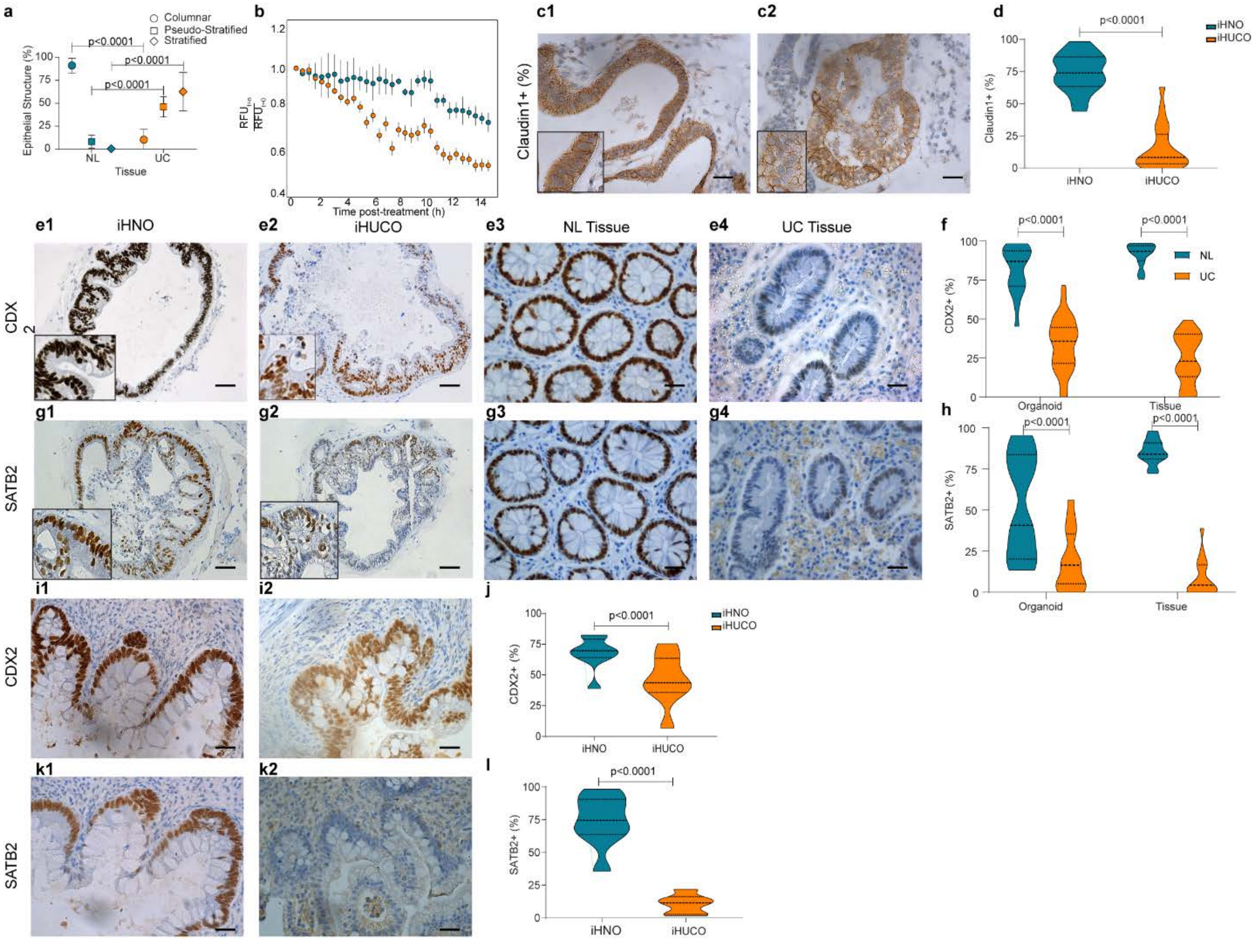

Supplementary Fig. 2: iHUCOs reveal a disruption in tight junctions and differentiation pattern as compared to iHNOs

a Percentage distribution of epithelial structure types -- columnar, pseudostratified, and stratified -- in the normal and UC matched primary tissues. The median value is indicated in the center of the box plot. The whiskers above and below the box plot represent 1 standard deviation above and below the mean of the data, respectively. **b** Epithelial barrier permeability measurements in real time for iHNOs (blue) and iHUCOs (orange) for 15 hours. Each independent biological sample was comprised 50-70 organoids pooled together per experiment for further analyses.**c, d** Representative IHC and quantification (shown as violin plots) for organoid cells expressing claudin-1. **e, g** Representative IHC for CDX2 and SATB2, demonstrating the lower expression of both proteins in UC and normal organoids with their matched primary tissues. **f, h** Violin plot of cells expressing CDX2 and SATB2 in iHNOs and iHUCOs and the primary tissues. **i, k** Representative IHC for CDX2 and SATB2 organoid-derived colon with **j, l** percentage distribution of cells expressing each protein, confirming a significant decrease in expression of CDX2 and SATB2 in UC vs. normal epithelium. An unpaired, non-parametric, two-sided Mann-Whitney *U* test was used for all statistical comparisons except for panel b, in which the non-

parametric Wilcoxon rank-sum test was used. Scale bar = 40 μm. n = 5 iHNOs and n = 6 iHUCOs except for barrier studies, n = 1. Source data are provided as a Source Data file.

Supplementary Figure 3 related to Figure 4

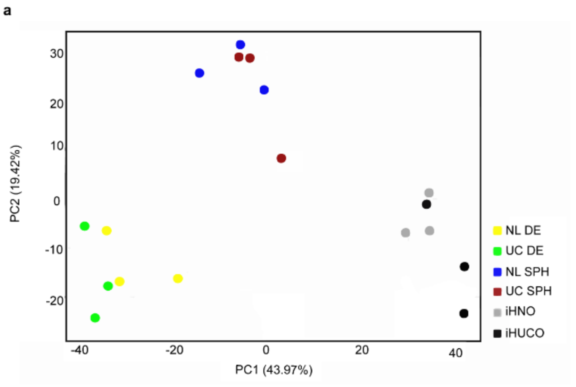

b

GO term Description	P-value	FDR q-value	Enrichment
Positive regulation of ion transport	6.34E-5	4.11E-3	2.40
Supramolecular fiber organization	1.16E-6	1.54E-4	2.07
Actin filament organization	8.19E-4	2.74E-2	2.05
G protein-coupled receptor signaling pathway	2.75E-5	2.13E-3	2.07
Regulation of interleukin-8 production	1.96E-4	9.66E-3	6.12
Interferon-gamma-mediated signaling pathway	2.28E-4	1.08E-2	2.95
Inflammatory response	1.01E-6	1.42E-4	3.01
Regulation of protein kinase B signaling	8.18E-6	7.36E-4	2.79
Response to mechanical stimulus	5.52E-6	5.24E-4	2.34
Integrin-mediated signaling pathway	8.72E-5	5.09E-3	3.68
Rho protein signal transduction	5.54E-4	2.02E-2	3.37
Cell-cell adhesion via plasma-membrane adhesion molecules	4.81E-4	1.84E-2	4.36

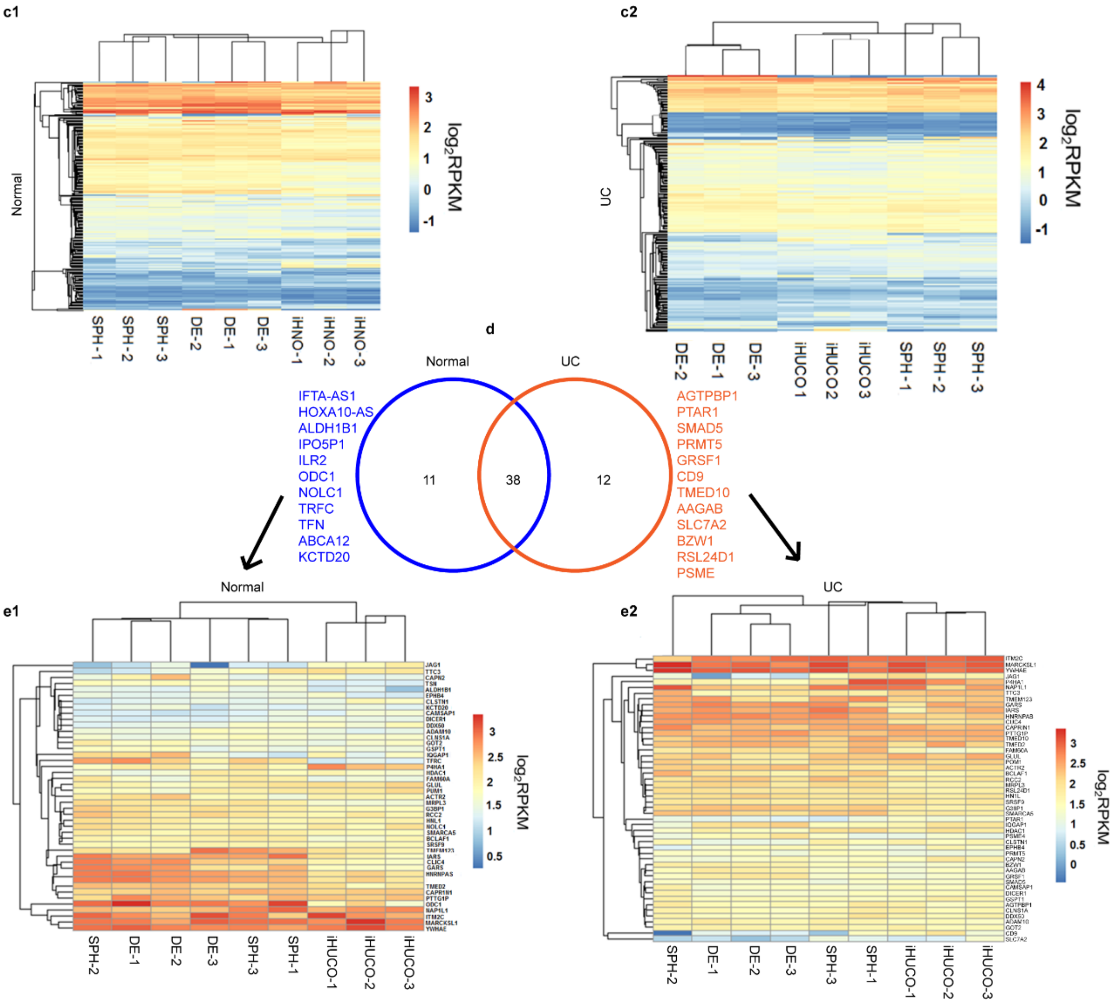

Supplementary Fig. 3: Bulk transcriptomic analysis of iHUCOs reveals the colonic identity. **a** Principal component analyses (PCA) conducted on normal and UC definitive endoderm (DE), spheroids (SPH), and organoids (n=3 each). The first principal component accounts for about 44% of the variation in the data. **b** Table of *P* values, FDR q-values, and enrichment scores for the enriched GO terms shown as REVIGO scatterplots[1] in Fig. 4e. **c** Heatmaps of the genes upregulated in colonic and small intestinal organoids and tissues reported by Munero et al[2].,

confirming the distinct developmental pattern in iHUCOs. **d** Venn diagram of the top 50 expressed genes in iHNOs and iHUCOs (extracted from the gene sets in panel c) with genes exclusive to normal (blue) or UC (orange). **e** Curated heatmaps for the top 50 genes in panel c to compare the normal and UC developmental signature.

Supplementary Figure 4 related to Figure 5

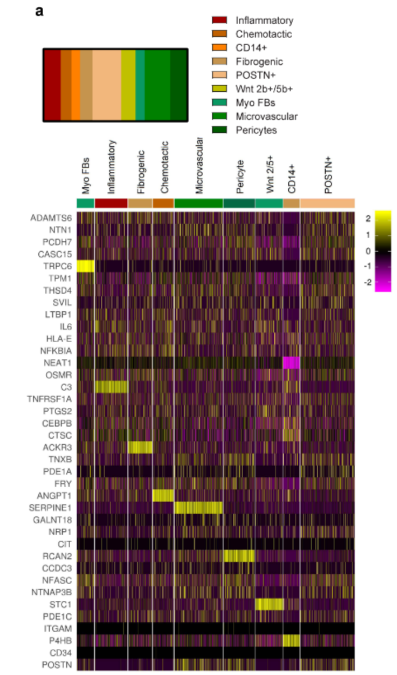

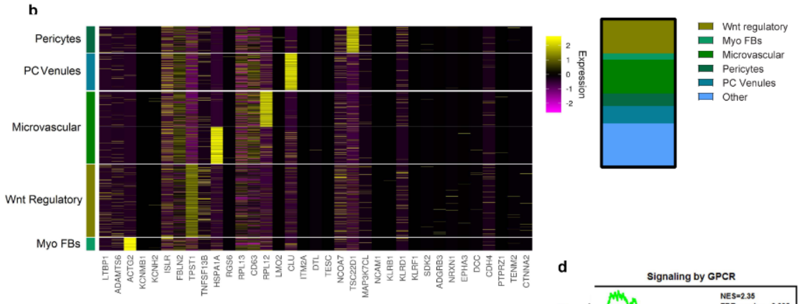

c

GO term Description	Enrichment	P-value
Angiogenesis	2.68	7.51E-04
Regulation of IL-6 production	9.05	3.55E-04
Neutrophil chemotaxis	32.49	2.43E-08
Myeloid leukocyte migration	21.94	1.68E-09
Regulation of dendritic cell differentiation	13.17	7.67E-05
Response to IL-1	8.12	2.36E-04
Activation of MAPK activity	2.96	2.50E-04
Positive regulation of inflammatory response	2.18	1.59E-04
G protein-coupled receptor signaling pathway	5.64	9.60E-06
Chemokine-mediated signaling pathway	27.07	1.44E-07
immune effector process	1.83	5.20E-07
antimicrobial humoral response	5.63	6.32E-07
type I interferon signaling pathway	3.01	3.01E-04

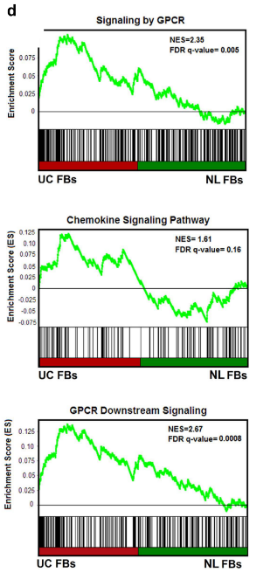

Supplementary Fig. 4 High-resolution transcriptomic analysis of the parental fibroblasts
a Proportion plot (top) and a heatmap (bottom) of highly expressed markers of the cell sub-populations in UC fibroblasts; Myo FBs = myofibroblasts; Wnt 2/5B+= Wnt2B+ and Wnt5B+ cells.. **b** Heatmap of highly expressed markers in normal fibroblast subsets with a proportion plot illustrating the cell sub-populations in normal fibroblasts; Myo FBs = myofibroblasts; PC venules= post-capillary venules. **c** Table of enrichment scores and *P* values for the GO terms presented as REVIGO scatterplots[1] in Fig. 5d. **d** Highly significant terms based on GSEA analysis of UC (red) vs. normal (green) fibroblasts, highlighting the role of GPCR and chemokine-mediated signaling in UC. Source data are provided as a Source Data file.

Supplementary Figure 5 related to Figure 6 & 7

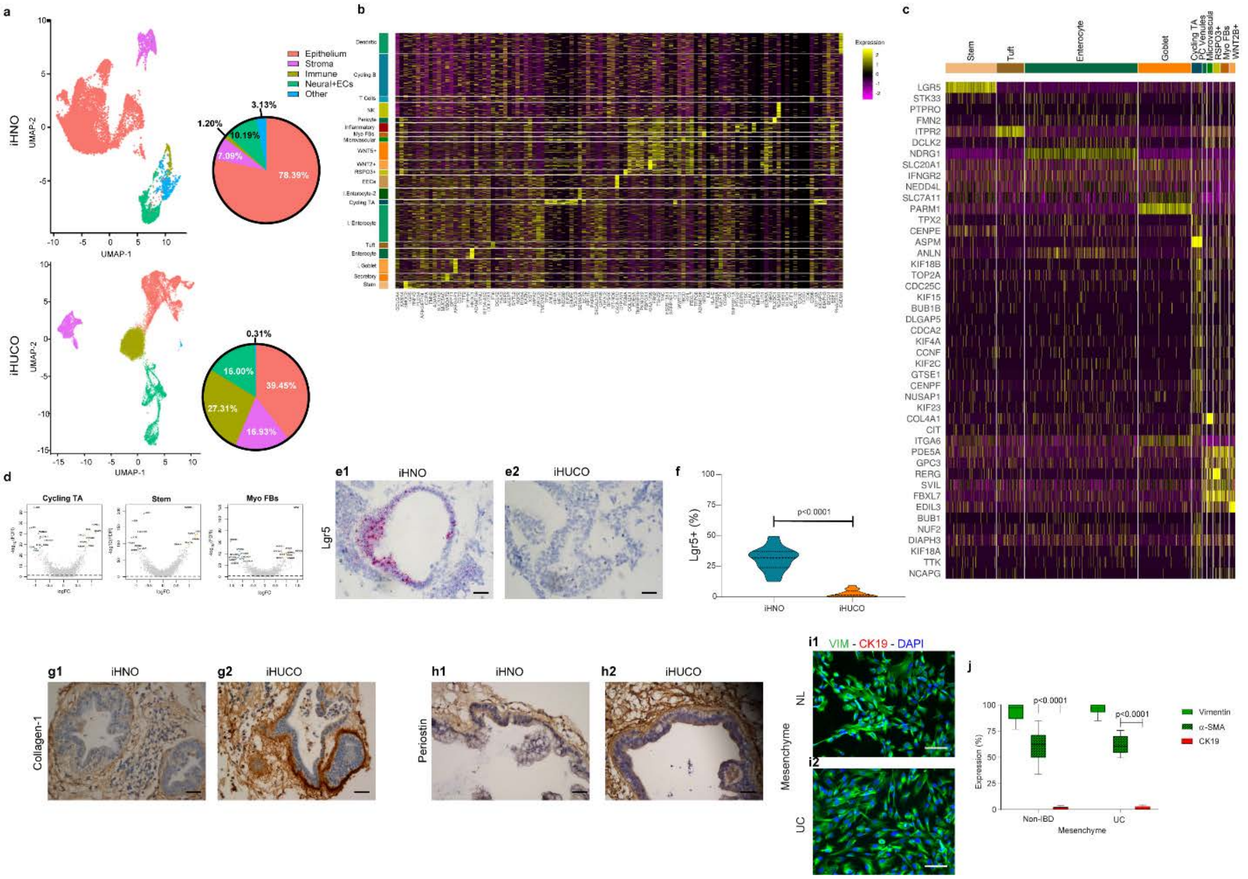

Supplementary Fig. 5 High-resolution transcriptomic analysis of iHNOs and iHUCOs

a UMAPs based on cell types in the organoids, highlighting 4 main cell types: epithelial, mesenchymal, immune, and neural + endothelial cells, with pie charts showing the proportion of each cell type in iHNOs and iHUCOs. Percentages of the indicated cell populations are indicated in the pie graphs.**b** Heatmap of highly expressed markers in each iHUCO epithelium and stroma subset. **c** Heatmap of highly expressed markers in iHNO epithelium and mesenchyme. **d,** Volcano plots for the selected subtypes highlight the highly significantly up- and down- regulated genes in iHUCOs **e** Representative in situ hybridization for Lgr5 in iHNOs and iHUCOs, with violin plots expressing Lgr5 in these organoids. **f.** A violin plot of the percentage of Lgr5+ cells in the iHNO compared to iHUCO.**g, h** Representative IHC for collagen type-1 and periostin in the organoids, highlighting the excessive production of both proteins in the stroma of iHUCOs when compared to iHNOs. **i** Representative IF staining of iHNO- and iHUCO-derived mesenchyme expressing vimentin (green) and lacking CK19 (red), with **j** summarized percentages of cells positive for vimentin, α-SMA (fibroblast markers), and CK19 (epithelium marker). The median value is indicated in the center of the box plot. The whiskers above and below the plot represent 1 standard deviation (SD) above and below the mean of the data, respectively.IF scale bar = 25μm. n = 5 iHNOs and n=6 iHUCOs, For comparisons in e and j, n unpaired, non-parametric, two-sided Mann-Whitney *U* test was used for all statistical comparisons. IHC scale bar = 40 μm. n=5 iHNOs (blue) and n=6 iHUCOs (orange). Source data are provided as a Source Data file.

Supplementary Figure 6 related to Figure 8 & 9

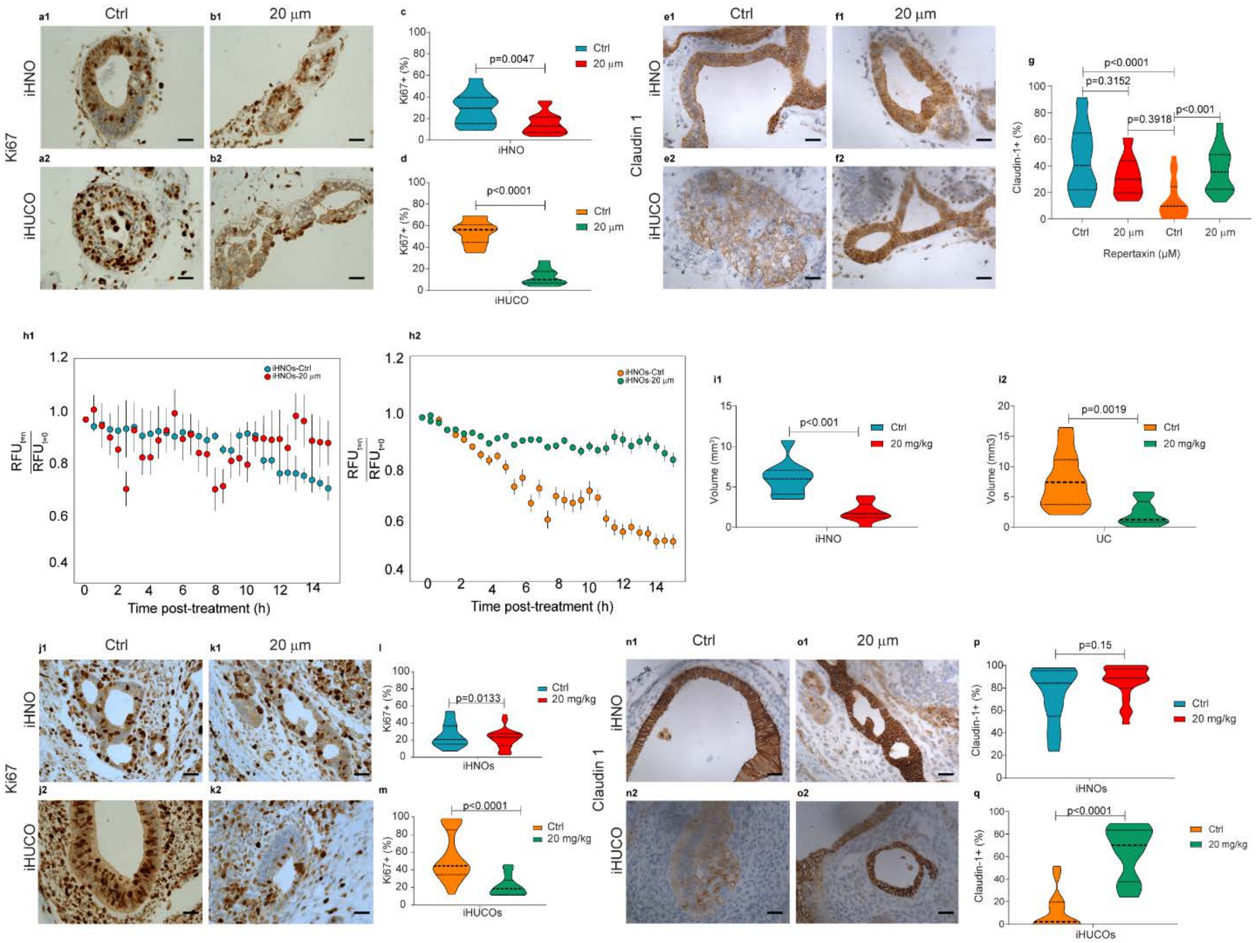

Supplementary Fig. 6: Repertaxin alters *in vitro* and *in vivo* the colitic signature in iHUCO epithelium

a-d Representative IHC for Ki67 in repertaxin-treated (20 µm) iHNOs and iHUCOs compared to control (Ctrl) organoids with percentage distribution of cells expressing Ki67 in all groups; **c**, **e, f** Representative IHC for claudin-1 in organoids with or without repertaxin treatment. **g** Violin plots showing the distribution of claudin-1-positive cells in repertaxin-treated and control organoids. **h** Relative epithelial barrier permeability to 4 kDa dextran measured in real time for control iHNOs (blue, n = 11) vs. repertaxin-treated iHNOs (red, n = 10) and control iHUCOs (orange, n = 17) vs. repertaxin-treated iHUCOs (green, n = 16) over 15 hours, demonstrating that repertaxin treatment significantly decreased iHUCO epithelial barrier permeability. Each independent biological sample was comprised 50-70 organoids pooled together per experiment for further analyses. **i** The calculated average volume (based on measurement with calipers) of the subcutaneously implanted organoids after 21 days with or without repertaxin injection was greater in control than repertaxin-treated organoids. **j, k, n, o** Representative IHC for Ki67 and claudin-1 in repertaxin-treated and control organoids after 21 days' treatment *in vivo*. **l, m, p, q** Violin plots of cells positive for Ki67 and claudin-1 in the presence or absence of repertaxin treatment *in vivo.* The unpaired, nonparametric Mann-Whitney U test was used for all comparisons excepting h1,2, in which the non-parametric Wilcoxon rank-sum test was used. The median value is indicated in the center of the box plot. The whiskers above and below the plot represent 1 standard deviation (SD) above and below the mean of the data, respectively. Scale bar = 40 µm. n= 5 iHNOs (blue) and n= 6 iHUCOs for a-g; n = 3 for j-q. Source data are provided as a Source Data file.

References

1. Supek F, Bosnjak M, Skunca N, Smuc T. REVIGO summarizes and visualizes long lists of gene ontology terms. *PloS one.* 2011;6(7):e21800.
2. Munera JO, Sundaram N, Rankin SA, et al. Differentiation of Human Pluripotent Stem Cells into Colonic Organoids via Transient Activation of BMP Signaling. *Cell Stem Cell.* 2017;21(1):51-64 e56.

Supplementary Table 1: Patient demographics

Patient ID	Disease	Pathology (overall)	M/F	Race	Disease Duration (Years)	Age at resection
CRL1541	Normal	Normal	F	NA	NA	22 weeks
NL-34	Cancer	Normal Margin	M	White	NA	70
NL-33	Cancer	Normal Margin	M	White	NA	62
NIBD -227	Cancer	Normal Margin	F	White	27	65
NIBD-304	Non-IBD	Normal	F	White	8	37
UC-5	UC	Active UC	M	White	2	20
UC-40	UC	Active UC	M	White	2	32
UC-35	UC	Active UC	F	White	30	47
UC-301	UC	Active UC	F	White	2	70
UC-303	UC	Active UC	M	White	4	44
UC-307	UC	Active UC	M	White	7	36

Supplementary Table 2: Short-tandem repeat fingerprinting

Patient ID	D13S317	D16S539	CSF1PO	TH01	vWA	D21S11	D7S820	D5S818	TPOX	AMEL
CRL1541	12	10,11	9,13	6,7	16,18	28,30	8,9	12	8	X
NL-34	11,12	9,12	11,12	9.3	14,17	29,30	9,12	11,12	8	X,Y
NL-33	11	11,12	11,12	8,9.3	15,17	29,32.2	10,12	9,11	8	X,Y
NL-227	11, 12	12, 14	10, 11	7, 9.3	14, 17	31	10	11, 12	8, 10	X
NL-304	12	11, 12	10, 11	7, 9	14, 17	29, 30	11	12	11, 12	X
UC-5	11	9,11	12	6,7	16,17	29,32.2	8	11,13	8,11	X,Y
UC-40	10,11	10	10,11	9.3	18,20	28,31	10,12	10,13	8,12	X,Y
UC-35	10,12	12	11,13	8,9.3	14,18	28,31	10,13	12,13	8,11	X
UC-301	11, 14	9, 11	11, 13	6, 7	15	28, 29	11, 12	11	8	X
UC-303	9, 10	11, 13	12	6, 9.3	17, 18	28, 29	11, 12	11, 12	8	X, Y
UC-307	12, 13	10, 12	10, 12	8, 9.3	16, 17	30, 32.2	8, 9	9, 12	8, 11	X, Y
