# Supplementary material for: Induced organoids derived from patients with ulcerative colitis recapitulate colitic reactivity
Source: Nat Commun. 2021 Jan 11;12:262. doi: 10.1038/s41467-020-20351-5 (PMC7801686; doi:10.1038/s41467-020-20351-5)
Supplement: Supplementary file 3 — Reporting Summary [file 41467_2020_20351_MOESM3_ESM.pdf]

nature research

Corresponding author(s): Emina Huang

Last updated by author(s): 2020-10 17

Reporting Summary

Nature Research wishes to improve the reproducibility of the work that we publish. This form provides structure for consistency and transparency in reporting. For further information on Nature Research policies, see our Editorial Policies and the Editorial Policy Checklist.

Statistics

For all statistical analyses, confirm that the following items are present in the figure legend, table legend, main text, or Methods section.

n/a	Confirmed	
☐	☒	The exact sample size (*n*) for each experimental group/condition, given as a discrete number and unit of measurement
☐	☒	A statement on whether measurements were taken from distinct samples or whether the same sample was measured repeatedly
☐	☒	The statistical test(s) used AND whether they are one- or two-sided<br>*Only common tests should be described solely by name; describe more complex techniques in the Methods section.*
☐	☒	A description of all covariates tested
☐	☒	A description of any assumptions or corrections, such as tests of normality and adjustment for multiple comparisons
☐	☒	A full description of the statistical parameters including central tendency (e.g. means) or other basic estimates (e.g. regression coefficient) AND variation (e.g. standard deviation) or associated estimates of uncertainty (e.g. confidence intervals)
☐	☒	For null hypothesis testing, the test statistic (e.g. *F*, *t*, *r*) with confidence intervals, effect sizes, degrees of freedom and *P* value noted<br>*Give P values as exact values whenever suitable.*
☒	☐	For Bayesian analysis, information on the choice of priors and Markov chain Monte Carlo settings
☒	☐	For hierarchical and complex designs, identification of the appropriate level for tests and full reporting of outcomes
☒	☐	Estimates of effect sizes (e.g. Cohen's *d*, Pearson's *r*), indicating how they were calculated

Our web collection on statistics for biologists contains articles on many of the points above.

Software and code

Policy information about availability of computer code

Data collection	DM IRB upright microscope-40X Leica (LAS X version 3.7.2)<br>Fiji http://fiji.sc versions 1.51d (June 2016) - 1.51w (March 2018)<br>Confocal microscope (DM16000) Leica
Data analysis	Fiji http://fiji.sc 1.51d (June 2016) - 1.51w (March 2018)<br>Prism-v8 graphing and statistical software GraphPad Software<br>FastQC v0.11.5/MultiQC v0.7 https://multiqc.info<br>STAR v2.5.3a https://github.com<br>DEseq2 v3.8 https://bioconductor.org<br>GSEA v2 http://software.broadinstitute.org<br>Cytoscape v3.5.1 https://cytoscape.org<br>RStudio v3.5.1/3, 3.6.2 and 3.5.3 https:/r-project.org<br>ggplot2 http://ggplot2.org March, 2019<br>Gorilla http://cbl-gorilla.cs, 2009<br>REVIGO http://revigo.irb.hr ; 2011<br>Gene Ontology Resource http://geneontology.org<br>Draw Venn Diagram http://bioinformatics.psb<br>Cutadapt v.1.10 DOI:10.14806/ej.17.1.200<br>Seurat 3.1.2<br>GraphPad Prism software v7 and v8<br>Adobe Illustrator CC2019 were used to design the final illustrations and supplementary figures. Microsoft word, Microsoft Excel (2015 - 2019

(Microsoft corporation), were used to generate the text and to store the original enumerated data.

For manuscripts utilizing custom algorithms or software that are central to the research but not yet described in published literature, software must be made available to editors and reviewers. We strongly encourage code deposition in a community repository (e.g. GitHub). See the Nature Research guidelines for submitting code & software for further information.

Data

Policy information about availability of data

All manuscripts must include a data availability statement. This statement should provide the following information, where applicable:

- Accession codes, unique identifiers, or web links for publicly available datasets
- A list of figures that have associated raw data
- A description of any restrictions on data availability

The next generation sequencing (NGS) datasets from this study have been deposited in the Gene Expression Omnibus repository (GEO), series accession number: GSE117345 and GSE152999. The weblinks are: https://www.ncbi.nlm.nih.gov/geo/query/acc.cgi?acc=GSE117345 and https://www.ncbi.nlm.nih.gov/geo/query/acc.cgi?acc=GSE152999, respectively. The reference transcriptome Hg19 (ftp://ftp.ensembl.org/pub/grch37/release-87/), KEGG (https://www.genome.jp/kegg/), and Reactome Pathways (https://reactome.org/) were used. The rest of data that support the findings of this study or further information and requests for reagents may be directed to the corresponding author upon reasonable request (huange2@ccf.org). Source data are available for figures 1h,j,l, 2b,d,f,h, 3c,e,g,i,k,m,o, 4,j, 7a,b,d,f,g, 8c,d,g,h,k,l,o,p,s,t, 9d,e,h,i,l,m,p,q,t,u, and Supplementary Information Figures 1b,f,h,j,l, 2a,b,d f,h,j, l, 4a,b, 5a,f,j, 6c,d,g,i,l,m,p,q

Field-specific reporting

Please select the one below that is the best fit for your research. If you are not sure, read the appropriate sections before making your selection.

☒ Life sciences ☐ Behavioural & social sciences ☐ Ecological, evolutionary & environmental sciences

For a reference copy of the document with all sections, see nature.com/documents/nr-reporting-summary-flat.pdf

Life sciences study design

All studies must disclose on these points even when the disclosure is negative.

Sample size	The study is aimed to define an approach to developing personalized models of ulcerative colitis containing autologous epithelial and mesenchyme. For the initial derivation, n = 3 for each primary diseased vs. normal phenotype was requested, including a commercially available source of fibroblasts (ATCC CRL1541). To expand the sample sizes for subsequent analyses, we increased these numbers to n = 6 for ulcerative colitis, and n = 5 for non-IBD. For the single nuclear analyses, Based on the sequencing depth and the abundance of cells in this scRNA-seq, sufficient statistical power has been achieved to detect differentially expressed genes and cell clusters. Here, we have 5 biological samples for normal condition and 6 biological samples for colitis condition. These yield a total number of cells around 174k. On average, we observed 23,871 number of reads per cell before normalization. We conducted simulation studies using state of the art power assessment tool POWSC [PMID: 32614380 DOI: 10.1093/bioinformatics/btaa607], which modeled expression level using a mixture of zero-inflated Poisson (ZIP) and log-normal Poisson (LNP) distributions. For small group comparison with only 2k cells per sample, the power will range from 0.788 to 0.985, with a marginal power of 0.922. The range of power comprehensively reflected the different strata of average reads. In all our single-cell analysis, we have achieved higher power, as the cell number is already much higher than the simulation setting.
Data exclusions	No data were excluded.
Replication	We selected 5-6 each biological replicates for each diseased vs. non-diseased tissue.For the derivation of the iHUCO and the iHNO, all biological replicates were successful. Phenotypes paralleled the primary disease. Therefore, for the morphology and immunohistochemistry evaluation, reprogramming, and directed differentiation, n = 5 for iHNO and n = 6 for iHUCO. For the single nuclear and in vivo experiments, n = 3 each for iHNO and iHUCO.
Randomization	The allocation was not random, we chose tissues based on the presence/absence of the disease and confirmed this histologically.
Blinding	Blinding was applied to enumeration and to pathological assessment. For the in vivo experiments using repertaxin, blinding was also applied to treatment (control vs. repertaxin) and growth measurements.

Reporting for specific materials, systems and methods

We require information from authors about some types of materials, experimental systems and methods used in many studies. Here, indicate whether each material, system or method listed is relevant to your study. If you are not sure if a list item applies to your research, read the appropriate section before selecting a response.

Materials & experimental systems

n/a	Involved in the study
☐	☒ Antibodies
☐	☒ Eukaryotic cell lines
☒	☐ Palaeontology and archaeology
☐	☒ Animals and other organisms
☐	☒ Human research participants
☒	☐ Clinical data
☒	☐ Dual use research of concern

Methods

n/a	Involved in the study
☒	☐ ChIP-seq
☒	☐ Flow cytometry
☒	☐ MRI-based neuroimaging

Antibodies

Antibodies used

Antibody Source Identifier
anti-α-SMA (SIGMA A5228 Clone 1A4, Lot Lot 074M4814V, dilution 1:200)
anti-CK19 (Abcam ab8187, Clone EP1508Y, dilution 1:100)
anti-Tra-1-60 (Abcam ab16288, dilution 1:500)
anti-Oct4 (R&D System AF1759, Lot JTW0208081, dilution 1:500)
anti-SOX17 (R&D System Part 967330, Lot KGA081041, dilution 1:10)
anti-Brachyury (R&D systems Part 967332, Lot KQPO315042, dilution 1:10)
anti-Otx2 (R&D systems Part 967331, Lot KNO0415041, dilution 1:10)
anti-FOXA2 [Santa Cruz Biotechnologies sc-6554 (discontinued) 1:100]
anti-CDX2 (Biogenex MU392A-UC, clone CDX2-88, dilution 1:10)
anti-Vimentin (R&D systems MAB2105, Clone 280618, dilution 1:100)
anti-Ki-67 (Novus Biologicals NB110-89717, Lot C1, dilution 1:1000)
anti-SATB2 (Abcam ab51502, clone KQPO315042, dilution 1:200)
anti-beta-Catenin (BD transduction Lab 610153, clone 14, dilution 1:200)
anti-E-Cadherin (R&D systems AF648, dilution 1:600)
anti-RhoA (Abcam ab54835, clone 1B12, dilution 1:100)
anti-CXCR1 (R&D systems MAB330, Clone 42705, dilution 1:800)
anti-CXCL8 (Abcam ab7747, dilution 1:100)
anti-CLDN1 (Abcam ab15098, Lot GR3248184-1, dilution 1:200)
anti-HLA-A (Abcam ab52922, Clone EP1395Y, Lot GR258732-26, dilution 1:200)
anti-Limch1 (Abcam ab96178, Lot GR15093-24, dilution 1:200)
Alexa Fluor 488 donkey anti goat (Thermo fisher scientific A11055, clone NA (polyclonal), dilution 1:600)
Alexa Fluor 488 donkey anti mouse (Thermo fisher scientific A21202, clone NA (Polyclonal), dilution 1:1000)
Alexa Fluor 568 goat anti rabbit (Thermo fisher scientific A11036 clone NA (Polyclonal), dilution 1:1000)
Alexa Fluor 568 donkey anti mouse (Thermo fisher scientific A10037 clone NA (Polyclonal), dilution 1:200)

Validation

Antibody Validation
anti-α-SMA (https://www.ncbi.nlm.nih.gov/pmc/articles/PMC7065809/; doi: 10.1371/journal.pone.0229445)
anti-CK19 (From manufacturer's website, knockout validated, also https://www.abcam.com/cytokeratin-19-antibody-ep1580y-cytoskeleton-marker-ab52625.html; Also https://www.nature.com/articles/s41467-020-15548-7#Sec9)
anti-Tra-1-60 (https://doi.org/10.1016/j.biomaterials.2010.07.031)
anti-4-Oct human (https://www.ncbi.nlm.nih.gov/pmc/articles/PMC5171803/)
anti-SOX17 (https://www.nature.com/articles/s41586-019-1654-9)
anti-Brachyury (https://www.nature.com/articles/s41586-019-1654-9)
anti-Otx2 (https://www.nature.com/articles/s41586-019-1654-9)
anti-FOXA2 (https://datasheets.scbt.com/sc-6554.pdf)
anti-CDX2 (http://store.biogenex.com/us/applications/ihc/controls/controls/anti-cdx-2-clone-cdx2-88.html)
anti-Vimentin (https://www.ncbi.nlm.nih.gov/pmc/articles/PMC5973531/)
anti-Ki-67 (https://www.ncbi.nlm.nih.gov/pmc/articles/PMC6461621/)
anti-SATB2 (https://www.ncbi.nlm.nih.gov/pmc/articles/PMC5413969/)
anti-beta-Catenin (https://www.ncbi.nlm.nih.gov/pmc/articles/PMC3207144/)
anti-E-Cadherin (https://www.ncbi.nlm.nih.gov/pmc/articles/PMC5358111/)
anti-RhoA (https://www.ncbi.nlm.nih.gov/pmc/articles/PMC4823002/)
anti-CXCR1 (https://www.ncbi.nlm.nih.gov/pmc/articles/PMC6370871/#ec0020)
anti-CXCL8 (https://www.ncbi.nlm.nih.gov/pmc/articles/PMC6651518/)
anti-CLDN1 (https://doi.org/10.1016/j.phrs.2020.104978)
anti-HLA-A (https://journals.plos.org/plosone/article?id=10.1371/journal.pone.0160882)
anti-Limch1 (https://www.sciencedirect.com/science/article/pii/S0925443917300546?via%3Dihub)

Eukaryotic cell lines

Policy information about cell lines

Cell line source(s)	CRL1541 was purchased from the ATCC. Colonic isolates from normal colon or colitic colon were retrieved when patients underwent surgical resections for either colon cancer (non-IBD tissues were retrieved from at least 10 cm away from the cancer), or from refractory colitis. In all cases, the patients' informed consent was obtained, and the study was approved by the investigational review board at the Cleveland Clinic (Cleveland, Ohio, USA).
Authentication	Short-tandem repeat was used to confirm the identity of CRL1541 comparing the STR to that on the ATCC website. For primary isolate validation, fibroblasts and subsequent derivatives, including iPSCs and organoids, underwent short-tandem repeat analyses and were compared to genomic DNA from either stored frozen tissues or from FFPE blocks from which sections and gDNA were extracted.
Mycoplasma contamination	All lines used in this study were routinely tested for mycoplasma infection and used only when negative.
Commonly misidentified lines (See ICLAC register)	*Name any commonly misidentified cell lines used in the study and provide a rationale for their use.*

Animals and other organisms

Policy information about studies involving animals; ARRIVE guidelines recommended for reporting animal research

Laboratory animals	NOD-SCID IL2 Gamma receptor null mice, Male and Female, 4 - 27 weeks old. Mice were housed in a barrier facility, with ad lib access to water and chow. Animals were 3-5 weeks old on supply, and were used at either 4 weeks for subcutaneous implantation studies, or at 12 weeks for the omental grafting procedures. The animals were identified by numbered cages and by ear punches. Ambient temperature was maintained at (ºF) 71 / 20 – 26 with humidity (%) 45-50.
Wild animals	No wild animals were used in the study.
Field-collected samples	No field-collected samples were used in the study.
Ethics oversight	Cleveland Clinic Foundation IACUC

Note that full information on the approval of the study protocol must also be provided in the manuscript.

Human research participants

Policy information about studies involving human research participants

Population characteristics

Patient ID	Disease	Pathology (overall)	M/F	Race	Disease Duration (years)	Age at resection
CRL1541	Normal	Normal	F	NA	NA	22 weeks
NL-34	Cancer	Normal	M	White	NA	70
NL-33	Cancer	Normal	M	White	NA	62
NIBD -227	Cancer	Normal	F	White	27	65
NIBD-304	Non-IBD	Normal	F	White	8	37
UC-5	UC	Active UC	M	White	2	20
UC-40	UC	Active UC	M	White	2	32
UC-35	UC	Active UC	F	White	30	47
UC-301	UC	Active UC	F	White	2	70
UC-303	UC	Active UC	M	White	4	44
UC-307	UC	Active UC	M	White	7	36

Recruitment	Patients were identified from the operating and case presentation logs of the department of colorectal surgery who were to undergo operative intervention for their disease process. Since patients were selected from those undergoing colectomy for chronic ulcerative colitis there may be selection and self-selection bias.
Ethics oversight	Case Western Reserve Institutional Review Board

Note that full information on the approval of the study protocol must also be provided in the manuscript.
