# Supplementary material for: Induced organoids derived from patients with ulcerative colitis recapitulate colitic reactivity
Source: Nat Commun. 2021 Jan 11;12:262. doi: 10.1038/s41467-020-20351-5 (PMC7801686; doi:10.1038/s41467-020-20351-5)
Supplement: Supplementary file 4 — Description of Additional Supplementary Files [file 41467_2020_20351_MOESM4_ESM.pdf]

Description of Additional Supplementary Files

Supplementary Data 1.

Key reagents, analytical software, and murine models. This file contains details regarding reagents used for immunostaining, growth factors, reprogramming and pluripotency validation kits, microscopy, analytical and imaging software, and murine model strain/vendor for the experiments.
