# Supplementary material for: Induced organoids derived from patients with ulcerative colitis recapitulate colitic reactivity
Source: Nat Commun. 2021 Jan 11;12:262. doi: 10.1038/s41467-020-20351-5 (PMC7801686; doi:10.1038/s41467-020-20351-5)
Supplement: Supplementary file 5 — Supplementary Data 1 [file 41467_2020_20351_MOESM5_ESM.pdf]

REAGENTS		
Immunostaining reagents/materials		
Donkey serum	EMD Millipore	S30-100ML
Goat Serum	mpbio	08642921
Triton X-100	SIGMA	T8787-50ML
Paraformaldehyde	SIGMA	P6148-500G
Donkey anti-goat Alexa fluor 488	Thermo Fisher Scientific	A11055
Donkey anti-mouse Alexa fluor 488	Thermo Fisher Scientific	A21202
Goat anti-rabbit Alexa fluor 568	Thermo Fisher Scientific	A11036
Donkey anti-mouse Alexa fluor 568	Thermo Fisher Scientific	A10037
VECTASHIELD mounting medium	VECTOR, Inc	ZC1216
CoverWell imaging chamber	ThermoFisher Scientific	C18160
Growth media and supplements		
DMEM	Gibco	
Fetal bovine serum (FBS)	R&D systems	S11150
hESC qualified Matrigel	BD Biosciences	354277
mTESR1 media	Stem Cell Technologies	05850
Matrigel	BD Biosciences	354234
RPMI Medium 1640	Thermo Fisher Scientific	11875
Penicillin-streptomycin (100 ×)	Thermo Fisher Scientific	15140-122
Defined fetal bovine serum (dFBS)	Hyclone	SH30070.02
Advanced DMEM:F12	Thermo Fisher Scientific	12634-010
L-glutamine (100 ×)	Thermo Fisher Scientific	25030-081
HEPES Buffer	Thermo Fisher Scientific	15630080
50 × B27 supplement w/o Vitamin A	Thermo Fisher Scientific	12587-010
N2 Supplement	Thermo Fisher Scientific	17502-048
Enzymes/growth factors		
Dispase	Thermo Fisher Scientific	17105-041

Activin A	Cell Guidance Systems	GFH6
recombinant human FGF4	R&D Systems	235-F4
CHIR99021	StemGent	04-0004-10
EGF	R&D Systems	236-EG
Noggin	R&D Systems	6057-NG
R-spondin 1	R&D Systems	4645-RS
Collagenase Type IA	Sigma-Aldrich	C9891
RIPA lysis buffer	Sigma-Aldrich	R0278
Protease inhibitors	Roche Diagnostics	0589791001
RNasin Plus Ribonuclease Inhibitors	Promega	N2615
SUPERaseIN RNase Inhibitor	Thermo Fisher Scientific	AM2696
Commercial Assays/kits/materials		
MycoAlert™ mycoplasma detection kit	Lonza	LT07-318
MycoAlert™ assay control Set	Lonza	LT07-518
CytoTune-IPS 2.0 reprogramming kit	ThermoFisher	A13780-02
Human Pluripotent Stem Cell Functional Identification Kit	R&D Systems	SC027B
RNAscope® 2.5 VS Assay-RED	Advanced Cell Diagnostics	322250
Dual-Glo® Luciferase Assay System	Promega	E2920
mRNA RED detection	Roche Diagnostics	7099037001
hu-PPIB probes	Advanced Cell Diagnostics	313909
bacterial dapB	Advanced Cell Diagnostics	312039
miRNeasy kits	Qiagen	217004
TruSeq Total RNA	Illumina	20020596
ThruPLEX® Illumina next-generation sequencing (NGS) library preparation	Rubicon	R400585
Amplification grade DNase I	Invitrogen	18068- 015
Human Cytokine Array C5	RayBiotech	AAH-CYT-5-2
Reparixin L-lysine salt	MedChemExpress	HY-15252

Thin wall glass capillaries	World Precision Instruments	TW100F-4
micropipette holder/tubing	Sutter Instruments	BR-MH2/ BR-AT
Mineral oil	Thermo Fisher Scientific	8042-47-5
4 kDa FITC-dextran	Thermo Fisher Scientific	D1844
Nuclei EZ Lysis Buffer	Sigma	N-3408
PluriStrainer 5/40/200 µm	PluriSelect	43-50005/040/200
Software/Tools		Versions
DM IRB upright microscope-40X	Leica	LAS X version 3.7.2
Confocal microscope (DM16000)	Leica	
Fiji	http://fiji.sc	1.51d (June 2016) – 1.51w (March 2018)
Prism-v8 graphing and statistical software	GraphPad Software	
FastQC v0.11.5/MultiQC v0.7	https://multiqc.info	
STAR v2.5.3a	https://github.com	
DEseq2 v3.8	https://bioconductor.org	
GSEA v2	http://software.broadinstitute.org	
Cytoscape v3.5.1	https://cytoscape.org	
RStudio v3.5.1/3	https:/r-project.org	V3.6.2 and 3.5.3
ggplot2	http://ggplot2.org	
Gorilla	http://cbl-gorilla.cs	2009
REVIGO	http://revigo.irb.hr	2011
Gene Ontology Resource	http://geneontology.org	
Draw Venn Diagram	http://bioinformatics.psb	
Cutadapt v1.10	DOI:10.14806/ej.17.1.200	
Seurat 3.1.2		
Adobe Illustrator	CC2019	
**Experimental Models: Organisms/Strains**		
NOD.Cg-Prkdc^{SCID} IL2rg^{tm1Wjl}/SzJ (NSG)	In-house breeding service (JAX lab)	
